# Carbonic Anhydrase II Deficiency: A Rare Case of Severe Obstructive Sleep Apnea

**DOI:** 10.3389/fped.2018.00213

**Published:** 2018-07-31

**Authors:** Emanuela di Palmo, Marcella Gallucci, Elena Tronconi, Rosalba Bergamaschi, Salvatore Cazzato, Claudio La Scola, Giampaolo Ricci, Andrea Pession

**Affiliations:** ^1^Department of Pediatrics, S. Orsola-Malpighi Hospital, University of Bologna, Bologna, Italy; ^2^Pediatric Emergency Unit, S. Orsola-Malpighi Hospital, University of Bologna, Bologna, Italy; ^3^Department of Mother and Child Health, Salesi Children's Hospital, Ancona, Italy

**Keywords:** osteopetrosis, obstructive sleep apnea, continuous positive airway pressure, acidosis, adenotonsillectomy, carbonic anhydrase II deficiency

## Abstract

The term osteopetrosis describes a group of rare hereditary diseases of the skeleton, characterized by an increase in bone density, caused by a defect in the development or function of osteoclasts. It comprises a clinically and genetically heterogeneous conditions ranging from infantile onset life-threatening forms to mildest adult onset forms. “Malignant” osteopetrosis is characterized by bone fragility, short stature, compressive neuropathies, hypocalcaemia, pancytopaenia. The deficiency of carbonic anhydrase II causes a moderate form, presenting classically as a triad of osteopetrosis, renal tubular acidosis (RTA), and cerebral calcification. This condition leads to specific craniofacial dysmorphisms associated with upper airway obstruction that may result in obstructive sleep apnea. Herein we report a case of osteopetrosis with RTA associated with severe OSAS successfully treated with continuous positive airway pressure (CPAP).

## Introduction

Obstructive sleep apnea syndrome (OSAS) is a frequent clinical manifestation associated with upper airway obstruction during sleep. Adenotonsillar hypertrophy plays a critical role in determining pediatric sleep-disordered breathing in children, but multiple anatomic obstructions should also be considered. An important predisposing factor in determining the severity of this condition might be craniofacial disharmony (1). Osteopetrosis is a rare heritable disorder that comprises a clinically and genetically heterogeneous group of conditions ranging from infantile onset life-threatening forms to mildest adult onset forms. Abnormalities in osteoclast differentiation or function leads to increased bone mass that can result in specific phenotypic features such as macrocephaly and facial or skeletal malformations.

The aim of this article is to present a case of osteopetrosis with OSAS and to review the literature to increase the awareness about this rare syndrome as a cause of severe OSAS.

## Case-report

A 4 year-old child was referred to our center for severe obstructive sleep apnea syndrome, failure to thrive and facial dysmorphisms. He was born at 37 weeks of gestation by operative delivery for oligoidroamnios in monochorionic bi-amniothic twin pregnancy. His birth weight was 2.325 grams. Parents were healthy and consanguineous (first degree cousin) coming from Tunisia (desert of Gafsa). His twin had undergone adenotonsillectomy for severe OSAS when he was 3 years old and as a complication of surgery he presented with an episode of severe respiratory distress which required transient tracheostomy.

The child was already followed elsewhere for psychomotor retardation. At the age of 18 months he underwent brain magnetic resonance imaging (MRI) which resulted negative. Karyotype, genetic analysis of Fragile X Mental Retardation 1 gene and Array-Comparative Genomic Hybridization were normal. When he was 3 years old he sustained a foot fracture.

A clinical examination revealed a child with slight body structure, plagiocephaly and brachycephaly, adenoidal facies, dysmorphic features with large and simplified ears, micro, and retrograthia, teeth malalignment, prominent forehead, prominent cheeks, pectus excavatum, digging of the last ribs. He had arched palate and a second degree tonsillar hypertrophy. His height was 96 cm (3^rd^ centile) and his weight was 12.2 kilograms (<3^rd^ centile). The neurological examination was normal.

Cardiovascular examination showed wide hyperdynamic beat, gallop rhythm (S4), marked S2 with a protosistolic murmur, hepatomegaly; at chest examination breath sounds audible with the noise transmitted from the upper airways.

He presented a severe condition of obstructive sleep apnea without important tonsillar hypertrophy confirmed by a positive oximetry with basal oxygen saturation of 93%, oxygen desaturation index (ODI) of 23.4/h, McGill Oximetry Scoring System (MOS) of 4 (2).

Polysomnogram moreover showed a reduction of Total REM Sleep Time (11.7%) compared to Total Sleep Time with apnea hypopnea index (AHI) of 11.3/h, obstructive apnea-hypopnea index (OAHI) of 10.2/h, central apnea index (CAI) of 1.1/h and a nadir of desaturation of around 60%.

Therefore he started nocturnal continuous positive airway pressure (CPAP) therapy at 6 cm H_2_0.

At the beginning the child refused to wear the CPAP mask, so minimal pressure was initially provided. In a few days CPAP adherence progressively improved, pressure was increased avoiding sleep apneas and the therapy was performed all night long.

**Figure 1:**
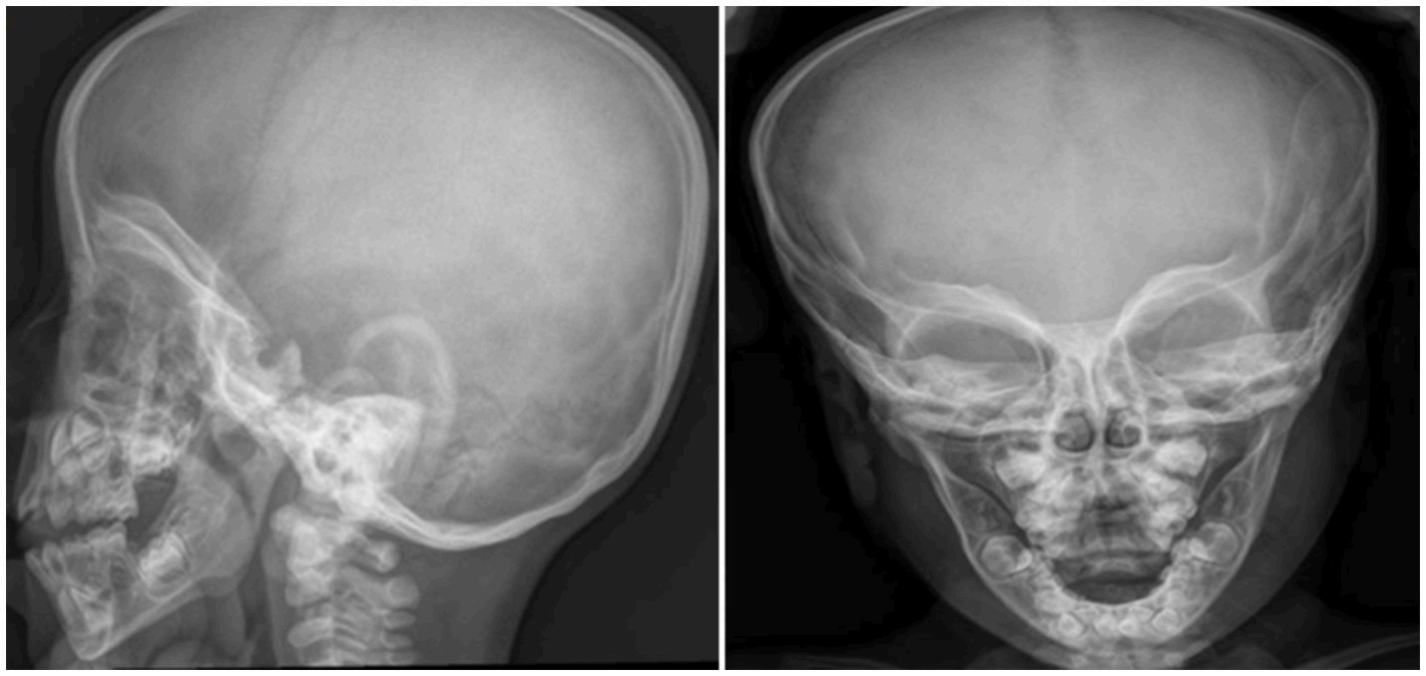
Cranial x-ray shows increased biparietal diameter, frontal bossing, “harlequin mask apparence” of orbits with increased density and thickness of the orbital roofs.

For the dysmorphic features he underwent cranial x-ray showing increased biparietal diameter with reference to frontal bossing, look to mask harlequin orbits characterized by increased density and thickness of the orbital roofs (Figure 1). Suspecting an osteo-thickening disease the x-ray was therefore extended to all the body. It confirmed the framework of alteration of bone density with little cortico-medullary differentiation in long bones and aspects like “bone to bone.”

Electrocardiography showed sinusal rhythm, right atrial hypertrophy, Katz-Watchel phenomenon due to balanced biventricular hypertrophy.

Echocardiography was performed detecting septal aspect characteristic of elevated pulmonary pression, dilated pulmonary artery, small atrial defect with left to right flow. No interventricular defects were demonstrable. The exam was positive for pulmonary hypertension in absence of congenital cardiac disease. The first clinical hypothesis was of pulmonary hypertension secondary to severe obstructive sleep apnea syndrome.

Renal ultrasound demonstrates an echogenic shadowing calculus of about 15 mm in diameter to the lower middle third of the left kidney.

The otorhinolaryngological assessment diagnosed only mild adenotonsillar hypertrophy in contrast to his severe OSAS. Conditioned orientation response audiometry (COR) revealed mild to moderate hearing loss.

Maxillo-Facial Computed Tomography (CT) was performed and the sagittal multiplanar reconstruction (MPR) images showed a reduction of the air spaces of nasopharynx.

Laboratory investigations showed mild iron deficiency anemia. Kidney and liver function, muscle enzymes, serum electrolytes, albumin, antitransglutaminase, Thyroid-Stimulating Hormone (TSH), Parathyroid Hormone (PTH), basal ammonium, and lactic acid were within the range of normality. Sweat test resulted normal. He had also vitamin D deficiency of 23.5 microg/L (30–100).

Arterial blood gas analysis showed metabolic acidosis with a pH of 7.20, ${\text{HCO}}_{3}^{-}$ 15.6 mmol/L, Base Excess −11.7. The presence of metabolic acidosis, urinary pH of 6.5, substantially preserved anion gap and renal stone, by excluding a gastrointestinal origin of acidosis, has guided us for a suspected distal Renal Tubular Acidosis (dRTA).

Oral therapy with sodium bicarbonate and potassium citrate was started with improvement of blood acidosis (Table 1) and blood ${\text{HCO}}_{3}^{-}$.

**Table 1:** Laboratory results in a child with severe obstructive sleep apnea and osteopetrosis with renal tubular acidosis.

	**Before medical treatment**	**Post medical treatment**
White blood cells (/mmc)	9.280	8.110
Hemoglobin (g/dl)	9.9	10.4
Red blood cells (/mmc)	4.410.000	4.420.000
Platelet count (/microL)	327.000	299.000
Blood urea (mg/dL)	39	50
Serum creatinine (mg/dL)	0.32	0.34
Serum sodium (mmol/L)	138	140
Serum potassium (mmol/L)	3.8	4.4
Serum chloride (mmol/L)	107	101
Serum calcium (mg/dL)	8.4	9.1
Arterial blood gas analysis pH at awakening	7.20	7.30
Arterial blood gas analysis pCO2 (mmHg) at awakening	55	47
Arterial blood gas analysis pO2 (mmHg) at awakening	80	85
Arterial blood gas analysis HCO^−^3 (mmol/L) at awakening	15.6	28.2
Base excess (mmol/L)	−11.7	2.3
Venous blood gas analysis pH	7.16	7.28
Venous blood gas analysis pCO2 (mmHg)	42	53
Venous blood gas analysis pO2 (mmHg)	61	56
Venous blood gas analysis HCO^−^3 (mmol/L)	15	24.9
Urine pH	6.5	8.5

**Figure 2:**
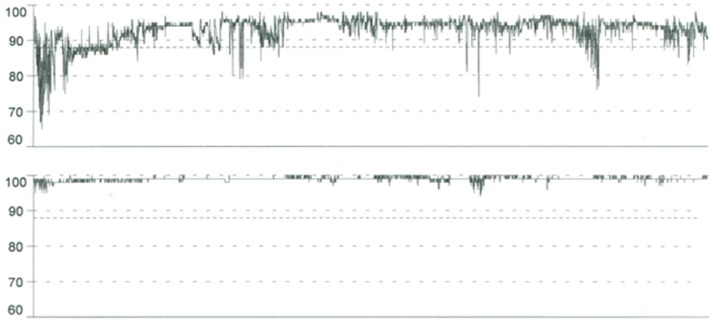
Pulse Oximetry tracings before CPAP (upper) shows basal SpO2 of 93%, SpO2 nadir of 65%, repetitive dips in oxygen saturation to < 90%; oxygen desaturation index (ODI) of 23.4/h. Pulse oximetry normalized with nocturnal CPAP treatment (below) in a child with severe obstructive sleep apnea and osteopetrosis with renal tubular acidosis.

A new pulse oximetry (Figure 2) and polysomnography (AHI: 0.6) during CPAP therapy was performed with a complete resolution of the apnoic events.

The CO_2_ values were monitored with arterial blood gas analysis without and with CPAP (pCO_2_: 56 mmHg and pCO_2_ 47 mmHg, respectively), as shown in Table 1.

Echocardiographic control showed normal pulmonary pressure and a left to right shunt through atrial septum; it was confirmed that the pre-existing pulmonary hypertension was secondary to severe OSAS and not primitive one. There was also an improvement of the height-weight growth.

Abdominal ultrasound was repeated detecting slight increase in kidney stone (17 mm) with ureteral obstruction and consequent upstream hydronephrosis (anteroposterior diameter of the pelvis in the axial scan of 27 mm). He underwent lombotomic pyeloplasty with concomitant removal of ipsilateral kidney stone.

The twin brother was suspected for OSAS, so he underwent to adenotonsillectomy at another hospital few months earlier without first investigating the severity of the sleep respiratory disorder.

Immediately after surgery, he had a severe respiratory failure and a tracheostomy and epigottoplasty were performed for marked malacia of the epiglottis. The traheostomy tube was removed after 2 months.

Subsequently, he came to our attention. The polysomnographic examination demonstrated a mild-moderate OSAS with AHI of 7.1/h, OAHI of 6.2/h and CAI of 0.9/h. He is still monitored over time evaluating the possibility of taking a treatment with non-invasive ventilation.

A clinical diagnosis of carbonic anhydrase II (CA-II) deficiency was made and a blood samples of all family members were collected for genetic testing. The molecular analysis revealed the presence of the c.232+1G>A homozigosis mutation (Exon 2: Sau3AI) in both twins, inherited from their carrier parents.

## Discussion

Osteopetrosis can be inherited as autosomal recessive, dominant, or X-linked traits. Classic or “malignant” autosomal recessive osteopetrosis is characterized by fractures, short stature, compressive neuropathies, hypocalcaemia, pancytopaenia.

A variant with renal tubular acidosis (RTA) is a moderate form, reported for the first time in 1972 (3, 4), caused by CA-II deficiency, identified as the main defect by Sly et al. in 1983 (5).

The clinical characteristics of this autosomal recessive syndrome are osteopetrosis, renal tubular acidosis and cerebral calcifications (6). Other clinical features include developmental delay, short stature, dental abnormalities, cranial nerve compression, history of multiple skeletal fractures, and cognitive defects varying from mild learning disabilities to severe mental retardation.

CAII is an enzyme present in renal tubular cells and osteoclasts. It plays a crucial role in renal regulation of acid/base homeostasis and in bone reabsorption by osteoclasts.

Osteoclasts are specialized cells, which degrade bone mineral and organic bone matrix, allowing the bone remodeling and the maintenance of bone biomechanical stability and mineral homeostasis.

Zinc-metalloenzyme carbonic anhydrase catalyzes the hydration of CO_2_ to form ${\text{HCO}}_{3}^{-}$ and H+ (7, 8). Protons are transferred into the extracellular environment by a proton pump. CAII have the highest catalytic activity so its deficiency interferes with the ability of osteoclasts to reabsorb bone.

Mechanism of RTA is based on cytosolic CA II deficiency. It does not determine the production of intracellular H+, which is secreted by a H+-ATPase in the tubular lumen to allow the reabsorption of bicarbonates (9).

This defect causes a mixed form of RTA due to waste of bicarbonates proximally and an incapacity to acidify the urine distally.

The CA II gene is located at q22 on chromosome 8. A total of 29 mutations distributed in almost all 7 exons and 4 introns of CAII have been described (10). These have an autosomal recessive inheritance pattern and consanguinity is a common feature in families with CA II mutations. CAII deficiency has been reported in several ethnic backgrounds, including Italian, German, French, Hispanic and African American but more than 70% of the cases have been described from the Arabian Peninsula (11, 12).

A *novel* splice junction at the 5′ end of intron 2 have been described by Hu et al. (12) as the common “Arabic” mutation. Patients of Arabic origin have a unique splice junction mutation at the junction of exon 2–intron 2 of the CA II gene (c.232 +1 G > A). The absence of fractures and the presence of mental retardation and high creatine phosphokinase (CPK) are suggestive of the peculiar Arabic variant of CA-II deficiency rather than other types of mutation.

The parents of the child we have described were first-degree cousin (children of stepbrothers) coming from the desert of Gafsa in Tunisia. Molecular analysis revealed the classical “Arabic” mutation.

Osteopetrosis, RTA, cerebral calcification, developmental delay, skeletal fractures (13), facial dysmorphism (14, 15), are common features in CA II deficiency. In our case is also present nephrocalcinosis and renal stones, conductive hearing loss, primary pulmonary hypertension, conditions less frequently described at the same time (15–18).

The first case of OSA occurring with malignant infantile osteopetrosis was described in 1988 by Carter et al (19). Stocks et al. documented that more than 50% of patients with malignant infantile osteopetrosis developed sleep apnea and sometimes tracheotomy was performed to manage OSA (20). On the contrary, severe OSAS has never been described in patients with osteopetrosis associated with renal tubular acidosis (CAII deficiency).

Like in autosomal dominant osteopetrosis, also in CAII deficiency otorinolaringoiatric complications should be carefully considered and dysmorphic features should be investigated before tonsillectomy. In these cases, the respiratory problems are due to the atypical growth of the bone and the craniofacial dysmorphic features. Therefore, medical treatment or adenotonsillectomy might not be resolutive or even dangerous then tracheostomy is sometimes required.

The craniofacial morphology of children affected by osteopetrosis, with hypognathism due to disordered growth of the mandible and decreased area between the base of the tongue and the posterior pharyngeal wall, can predispose to OSA. In addition, the bone involvement of the ribs causes restrictive changes of the chest wall that worsens lung ventilation.

OSAS is highly prevalent in children with other heterogeneous craniofacial conditions associated with upper airway obstruction: syndromic craniosynostosis (Apert, Cruzon, Pfeiffer, Nuenke, and Saerthe-Chozen syndromes), Down syndrome, achondroplasia, cleft palate and syndromes associated (Stickler syndrome, Goldenhar syndrome, Nager syndrome) (21), hemifacial macrosomia and conditions with micrognathia (Pierre Robin sequence, Treacher Collins syndrome).

It is known that children with untreated and prolonged OSAS, are at risk of severe health problems, including failure to thrive and cardiovascular diseases such as hypertension, cor pulmonale and left ventricular hypertrophy (22). The diagnosis and treatment of this condition should therefore be timely.

Therefore in these patients a careful evaluation of entity and etiopathogenesis of the respiratory disorder is fundamental to estimate the risk/benefit ratio of a possible adenotonsillectomy.

As matter of fact, the twin brother presented an episode of respiratory failure post-adenotonsillectomy that required tracheostomy.

On the contrary, in our case the presence of severe apnea unexplained by mild tonsillar hypertrophy led us to prefer the non-invasive approach with nocturnal CPAP instead of an intervention that could have put the patient's life at risk.

Moreover, the early use of nocturnal CPAP had reversed the cardiologic complications; after 2 months pulse oximetry parameters improved and after 6 months of therapy we observed also a gain in the height-weight growth.

## Concluding remarks

CAII deficiency is a clinical diagnosis based on the presence of acidosis and intracerebral calcifications together with typical radiological signs; confirmed by molecular testing. This condition, although rare, should be considered a possible cause of severe OSAS associated with craniofacial dysmorphic features.

The article also focuses on the usefulness of an integrated multidisciplinary approach to the management of these children at higher risk for complications related to OSAS.

## Ethics statement

Written informed consent for the publication of this case report and figures were obtained from the parents.

## Author contributions

EdP collected data and wrote the manuscript, MG, ET, RB, SC, and CL collected data, GR and AP reviewed the article.

### Conflict of interest statement

The authors declare that the research was conducted in the absence of any commercial or financial relationships that could be construed as a potential conflict of interest.
